# Pericardial computed tomography imaging findings in the setting of coccidioidomycosis

**DOI:** 10.1186/s12879-022-07601-1

**Published:** 2022-07-17

**Authors:** Mohammad H. Madani, Ahmadreza Ghasemiesfe, Yasser G. Abdelhafez, Lorenzo Nardo

**Affiliations:** Department of Radiology, University of California Davis, 4860 Y Street, Suite 3100, Sacramento, CA 95817 USA

**Keywords:** Coccidioidomycosis, Pericardium, Imaging, Computed tomography

## Abstract

**Background:**

Pericardial disease can be a manifestation of infection and imaging can have a role in its diagnosis. coccidioidomycosis endemic fungal infection has been more frequently reported over the past few decades. Other than case reports or series, there has been no systemic study evaluating pericardial imaging findings in patients with coccidioidomycosis to the best of our knowledge. The purpose of this study was to evaluate intrathoracic computed tomographic (CT) imaging abnormalities in patients with coccidioidal infection with specific emphasis on the pericardium.

**Methods:**

Retrospective review of radiology reports and clinical chart review was performed to identify patients with coccidioidomycosis between January 2000 and September 2021 at our medical center. Diagnosis of infection was confirmed predominately with serology. Patients were excluded if a CT was not performed within 3 months of confirmed diagnosis date and if there was concomitant additional granulomatous or fungal infection. Chest CT was reviewed for pericardial and additional intrathoracic findings.

**Results:**

The final retrospective cohort consisted of 37 patients. Imaging findings included lung nodules (N = 33/37), consolidation (N = 25/37), mediastinal or hilar lymphadenopathy (N = 20/37) and pleural effusions (N = 13/37). Eleven of 37 patients (30%) had either trace pericardial fluid (N = 3/37) or small pericardial effusions (N = 8/37). One patient had pericardial enhancement/thickening and history of pericardial tamponade. No other patient had clinical pericarditis or pericardial tamponade. Pericardial calcifications were not seen in any patient. Pericardial effusion was statistically associated with presence of pleural effusion as 9/13 patients with pleural effusion had pericardial effusion versus 2/26 patients without pleural effusion had pericardial effusion (p < 0.001). Otherwise patients with and without pericardial imaging findings were similar in terms of demographics, comorbidities and other imaging findings.

**Conclusion:**

Pulmonary parenchymal pathology is a common manifestation of coccidioidal infection. Most patients with coccidioidomycosis do not have pericardial imaging abnormalities on CT.

## Background

Coccidioidomycosis is a fungal infection that is endemic in Southwest United States including areas in California, New Mexico, Arizona and Texas [1] as well as Mexico and Central America [2]. The infection results from inhalation of spores of the Coccidioides species [1]. Sixty percent of individuals with infection are asymptomatic [2]. Clinical symptoms associated with infection include fever, cough, chest discomfort, fatigue or headache and are usually self-limited, lasting up to 3 weeks [3].

In the clinical setting the diagnostic imaging work up usually starts with chest radiographs. However, given the low sensitivity of radiographs, CT is considered the next imaging modality by American College of Radiology (ACR) appropriateness criteria in the appropriate clinical setting of respiratory illness given its higher sensitivity for thoracic findings of infection [4]. CT allows for optimal evaluation of infection including lung consolidation, nodules, intrathoracic lymphadenopathy and pleural effusion occuring in the setting of acute pulmonary coccidioidomycosis [5]. Residual lung consolidation, nodules, lymphadenopathy and pleural effusion may be seen with chronic infection [5]. Rarely, extrapulmonary disseminated infection can occur [1]. Management with antifungal therapy may be influenced by disease status and severity [1]. Meningitis, osteomyelitis, soft tissue infection and skin lesions are extrapulmonary sites of disseminated disease that have been reported [6].

The pericardium surrounds and provides structural support to the heart [7]. Disease of the pericardium may manifest as pericarditis, pericardial constriction, tamponade with potential morbidity and mortality [8, 9]. Pericarditis may result from both infectious or non-infectious etiologies. Of the infectious etiologies, pericarditis is frequently due to viral infection in developed countries while tuberculosis commonly accounts for majority of cases in developing countries [10]. Imaging can play a supportive role in diagnosis of pericardial disease. Pericardial effusion is considered a potential criterion for diagnosis of pericarditis in addition to clinical, physical exam and electrocardiographic criteria [11, 12]. Imaging features of pericardial thickening, enhancement and calcification can also be seen with pericardial disease [13, 14].

There have been published case reports regarding pericardial involvement by coccidioidomycosis or review of such case reports [15–17] and lack of systematic studies focused on imaging features of the pericardium in the setting of coccidioidal infection. For example, a literature review of published case reports or series spanning the years 1966 to 2003 found that an enlarged cardiac silhouette on chest radiograph was noted in ten of the seventeen patients with coccidioidal pericarditis [17]. However, the sensitivity of detection of pericardial effusion by radiography is inferior to CT imaging. Additionally, CT is advantageous compared with radiography with regards to its ability to detect pericardial thickening, enhancement and calcification. To the best of our knowledge, our study is the first and largest study from a single institution focused on investigating pericardial CT imaging findings in patients with coccidioidomycosis.

## Methods

### Patients

This is a retrospective chart and imaging review study. The University of California Davis Office of Research Institutional Review Board Administration provided ethical approval for this study and waived informed consent due the retrospective nature of the study. Inclusion criteria were adult patients (> 18 year old) with a confirmed diagnosis of coccidioidomycosis by serology, culture, or biopsy, and a chest CT, with or without contrast, performed within 3 months from the established diagnosis date. If multiple CT scans were performed, the one closest to the established diagnosis date was considered. Patients with other granulomatous or fungal infections such as tuberculosis and aspergillus were excluded.

Our aim was to investigate specifically CT imaging characteristics of the pericardium in patients with coccidioidal infection. We chose CT as our modality of interest as other modalities such as MRI were not performed and echocardiography was variably performed for our patients. Chest radiography was not utilized as CT is a more sensitive imaging modality for evaluation of the pericardium. Nuance mPower Clinical Analytics software (Nuance Communications, Inc, Burlington, MA, USA) was utilized to query the radiology reports corresponding with chest CT, performed between January 2000 to September 2021, containing the keyword “cocci” to capture variations of this type of infection.

A total of 54 unique records were initially identified; 17 patients were subsequently excluded due to an evidence of concurrent additional granulomatous or fungal (n = 5) infection; or long interval between CT and diagnosis (n = 10). Two children were additionally excluded, leaving a valid cohort of 37 patients; 10 females, 27 males, with mean age of 50.9 ± 15.3 years (range: 26–80 years).

For the majority (34/37; 92%) of patients, the diagnosis of coccidiomycosis was established based on serology (immunodiffusion/complement fixation). Remainder of patients were diagnosed via lung parenchymal or intrathoracic lymphadenopathy biopsy (n = 2), or pleural fluid culture (n = 1).

The patient’s characteristics, symptoms, medical comorbidities, and smoking history was extracted from the electronic medical record. Acute disease was defined as 1–3 weeks from onset of symptoms and chronic as greater than 3 weeks from the onset of symptoms. Involvement of additional organ systems or extrapulmonary sites of infection such as the central nervous system, skin or bones were also noted.

### Image analysis

Imaging in the form of CT of the chest, with (n = 20) and without (n = 17) intravenous contrast administration, was directly reviewed on picture archiving and communication system (PACS) by a board-certified cardiothoracic fellowship trained radiologist who was blinded to the clinical data at the time of imaging evaluation. Thoracic imaging was evaluated for abnormalities such as consolidation, masses, nodules, intrathoracic lymphadenopathy, pericardial effusion, pericardial calcification, pericardial enhancement, thickening and pleural effusion. Pulmonary lesions larger than 3 cm in size were considered pulmonary mass and those less than 3 cm as nodules. Pulmonary consolidation was defined as homogenous increased parenchymal attenuation that obscures vessels and airway walls. Mediastinal or hilar lymphadenopathy was defined as lymph nodes measuring greater than or near 1 cm in short axis diameter. Pericardial calcification, thickening and enhancement were determined by visual assessment as present or absent with enhancement assessed for contrast enhanced chest CTs only. Pericardial and pleural effusions were also graded into trace, small or large by visual assessment. Echocardiography findings were extracted from the clinical echocardiogram report.

### Statistical analysis

The primary endpoint was chest CT evidence of pericardial involvement (effusion, calcification, and/or thickening). Comparison between the two sub-groups with and without pericardial involvement regarding the different clinical and radiological features was performed via Fisher’s exact test for categorical variables (such as sex, risk factors) and Mann–Whitney U test for continuous variables (such as the age). Correlation between two ordinal values (such as the volumes of pleural and pericardial effusions) was calculated using Spearman’s correlation coefficient (Rho) with bootstrapping over 1000 samples.

Data was summarized as mean and standard deviation or frequency and percentage as appropriate. In all tests, a two-tailed p-value less than 0.05 was considered statistically significant. All analyses were performed using SPSS version 21 (IBM Corp, Armonk, New York, USA).

## Results

### Patients

A total of 11 patients with pericardial findings were identified, while the remaining 26 patients did not show CT evidence of pericardial findings. The general demographic and clinical characteristics of all the study patients is summarized in Table 1.

**Table 1:** Demographic and clinical characteristics of 37 patients with cocci subgrouped by the positive pericardial findings positivity on CT chest

Characteristic		Total	Pericardial findings on CT N, %		
			No	Yes	p-value
Age, mean ± SD (min–max)		50.9 ± 15.3 (26–80)	49.2 ± 15.6 (26–78)	54.2 ± 14.8 (26–80)	0.42
Sex	Female	10	5, 19%	5, 45%	0.12
	Male	27	21, 81%	6, 55%	
Race	White	21	15, 58%	6, 55%	0.31
	Hispanic	11	7, 27%	4, 36%	
	Black	1	0, 0%	1, 9%	
	Asian	4	4, 15%	0, 0%	
Smoking history	No	22	14, 54%	8, 73%	0.47
	Yes	15	12, 46%	3, 27%	
Comorbidities	No	12	9, 35%	3, 27%	1.00
	Yes	25	17, 65%	8, 73%	
DM	No	25	19, 73%	6, 55%	0.44
	Yes	12	7, 27%	5, 45%	
Autoimmunity	No	32	23, 88%	9, 82%	0.62
	Yes	5	3, 12%	2, 18%	
Cancer	No	33	23, 88%	10, 91%	1.00
	Yes	4	3, 12%	1, 9%	
Obesity	No	35	24, 92%	11, 100%	1.00
	Yes	2	2, 8%	0, 0%	
Clinical presentation					
Asymptomatic	No	35	24, 92%	11, 100%	1.00
	Yes	2	2, 8%	0, 0%	
Fever	No	21	15, 58%	6, 55%	1.00
	Yes	16	11, 42%	5, 45%	
Cough	No	20	13, 50%	7, 64%	0.50
	Yes	17	13, 50%	4, 36%	
Fatigue or SOB	No	30	24, 92%	6, 55%	0.02
	Yes	7	2, 8%	5, 45%	
Weight loss	No	34	23, 88%	11, 100%	0.54
	Yes	3	3, 12%	0, 0%	
Pain (chest, abd., back)	No	27	20, 77%	7, 64%	0.44
	Yes	10	6, 23%	4, 36%	
Hemoptysis	No	34	23, 88%	11, 100%	0.54
	Yes	3	3, 12%	0, 0%	
Chronicity	Acute	15	9, 35%	6, 55%	0.30
	Chronic	22	17, 65%	5, 45%	
Other organs (any)	No	31	23, 88%	8, 73%	0.34
	Yes	6	3, 12%	3, 27%	
CNS	No	33	25, 96%	8, 73%	0.07
	Yes	4	1, 4%	3, 27%	
Bone	No	33	24, 92%	9, 82%	0.57
	Yes	4	2, 8%	2, 18%	
Liver	No	36	25, 96%	11, 100%	1.00
	Yes	1	1, 4%	0, 0%	

Except for age, data shown as number and percentage within columns (N, %)

There was no significant difference regarding the demographics (age, sex, race) and duration of infection between patients with and without pericardial findings. Also, smoking history and potential risk factors of these two subgroups were comparable

Medical comorbidities were noted: autoimmune disease consisted of rheumatoid arthritis (n = 2), systemic juvenile idiopathic arthritis (n = 1), celiac disease (n = 1), and ulcerative colitis (n = 1). Malignancies included renal cell carcinoma (n = 1), rectal carcinoma (n = 1), acute myeloid leukemia (n = 1), and papillary thyroid carcinoma (n = 1).

Clinical symptoms that patients experienced were mostly cough (46%, n = 17) and fever (43%, n = 16). Shortness of breath and chest pain were the next most reported symptoms (both 19%, n = 7). Other less commonly reported symptoms included abdominal pain (3%, n = 1) and back pain (3%, n = 1). Two of the 37 patients were asymptomatic.

Duration of infection was chronic for most patients (22/37, 59%) and acute for 15 of 37 patients (41%).

Extrapulmonary manifestations of infection were seen in a total of 6 patients and consisted of central nervous system in 4/37 patients (8%), musculoskeletal abnormalities in 4 patients (8%), and hepatic involvement in 1/37 patients (3%).

### Chest CT findings

**Fig. 1:**
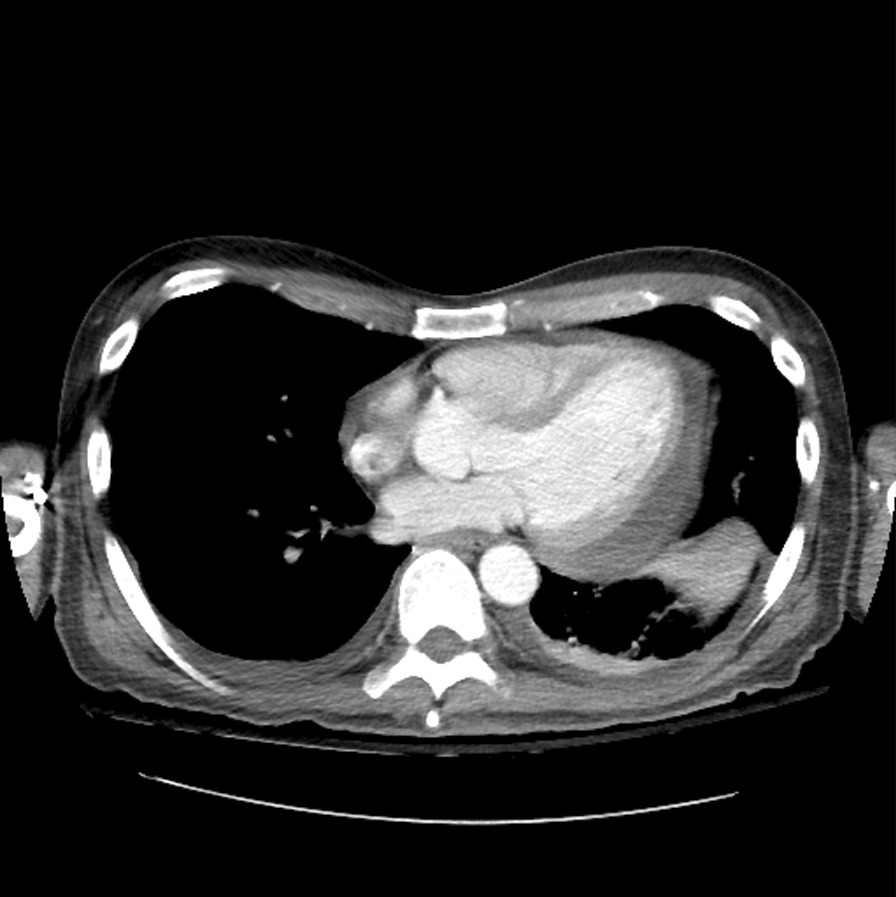
51 year-old male with chronic presenting with shortness of breath. Axial CT image at the level of the heart demonstrates a small pericardial effusion with thickening/enhancement, trace pleural effusions and left basilar consolidation

**Fig. 2:**
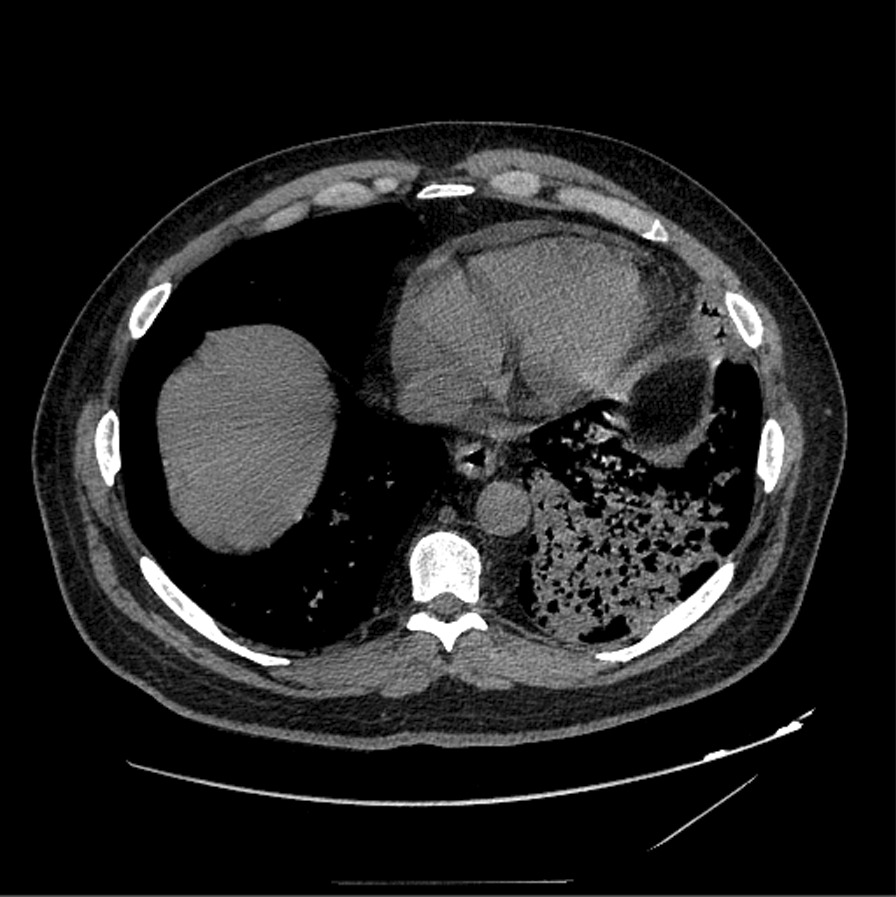
44 year old male with acute coccidioidomycosis presenting with fever, cough, and shortness of breath. Axial CT image at the level of the lower chest demonstrates a small pericardial effusion with extensive consolidation at the left lung base

**Fig. 3:**
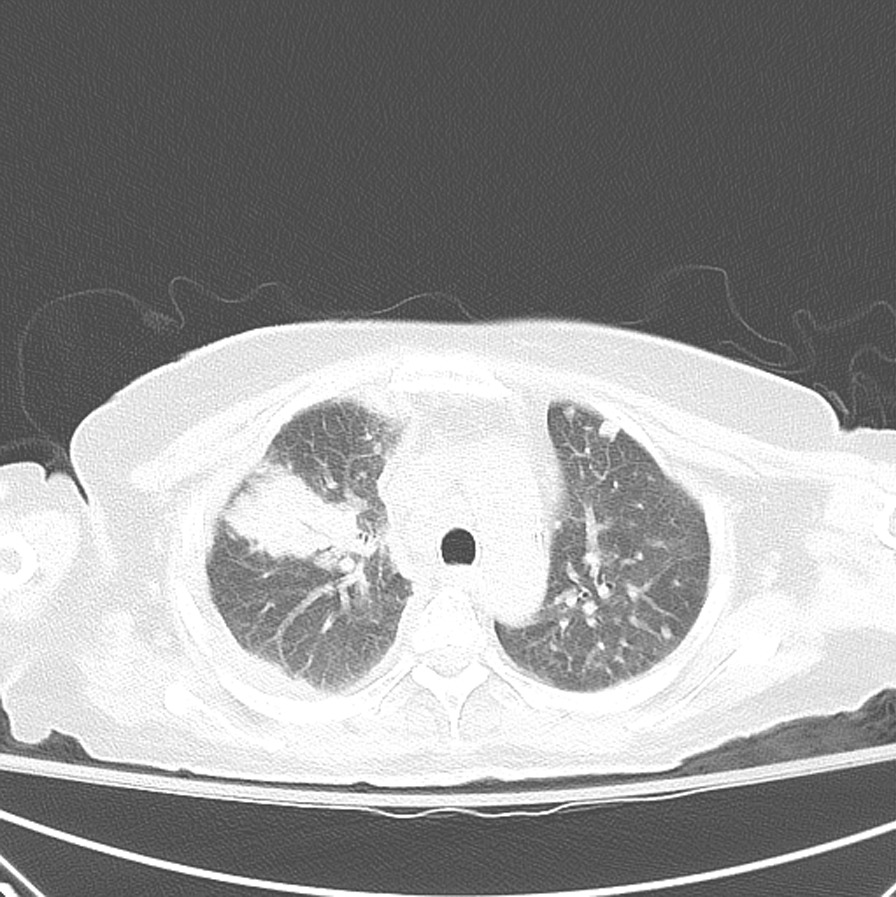
60 year-old female with acute coccidioidomycosis presenting with shortness of breath and chest pain. Right upper lobe consolidation, bilateral lung nodules, and partially imaged right pleural effusion are shown on this axial CT image of the lungs

Overall CT imaging features are summarized in Table 2. Representative CT images are shown in Figs. 1, 2 and 3.

**Table 2:** Thoracic CT imaging features subgrouped by pericardial CT findings

Thoracic CT chest features		Total	Pericardial findings on CT N, %		
			No	Yes	p-value
Parenchymal changes					
Nodules	No	4	2, 8%	2, 18%	0.57
	Yes	33	24, 92%	9, 82%	
Miliary pattern	No	30	22, 85%	8, 73%	0.40
	Yes	7	4, 15%	3, 27%	
Consolidation	No	12	11, 42%	1, 9%	0.06
	Yes	25	15, 58%	10, 91%	
Cavitation	No	24	18, 69%	6, 55%	0.46
	Yes	13	8, 31%	5, 45%	
Mass(s)	No	33	23, 88%	10, 91%	1.00
	Yes	4	3, 12%	1, 9%	
Mediastinal/hilar LNs	No	17	14, 54%	3, 27%	0.17
	Yes	20	12, 46%	8, 73%	
Pleural effusion	No	24	22, 85%	2, 18%	< 0.001
	Yes	13	4, 15%	9, 82%	
Laterality	Unilateral	7	4, 100%	3, 33%	0.07
	Bilateral	6	0, 0%	6, 67%	
Volume	Trace	6	2, 50%	4, 44%	1.00
	Small	6	2, 50%	4, 44%	
	Large	1	0, 0%	1, 11%

All patients had evidence of pulmonary parenchymal abnormalities on CT. The most prevalent CT involvement pattern was nodule formation seen in 33 out of 37 (89%) patients. This involvement was solitary in 1 patient and multiple in 32 patients. A miliary pattern of lung nodules occurred in 7 of 37 patients (19%). Cavitation within the nodules was seen in 2 patients.

Lung consolidation was the second most prevalent pattern, seen in 25 of 37 patients (68%). Consolidation was associated with cavitation in 8 patients.

Intrathoracic lymphadenopathy including mediastinal or hilar lymphadenopathy was present in 54% of patients (20/37).

Thirty five percent of patients (13/37) had pleural effusions with most of them having trace (n = 6) or small (n = 6) pleural effusions. Only one patient had a large pleural effusion. Pleural effusion was on the left side (n = 1), right side (n = 6) or bilateral (n = 6).

### Pericardial findings

Twenty-six out of 37 (70%) did not have pericardial findings. Trace or small pericardial effusions were noted in 3 and 8 patients, respectively. One of 11 patients with pericardial effusions had concomitant pericardial thickening/enhancement and had previously undergone pericardiocentesis for pericardial tamponade. However, 7 of 11 patients with pericardial effusions had noncontrast CT scans. Pericardial culture for the patient who underwent pericardiocentesis revealed no signs of infection. None of the other patients with pericardial effusions had clinical signs of pericardial tamponade or pericarditis. Pericardial calcifications were not observed in any of the patients.

Pericardial effusion was strongly associated with the presence of pleural effusion. Nine of 13 patients (69%) with pleural effusion also demonstrated pericardial effusion; compared to 2/26 (8%) without pleural effusion (P < 0.001). Pleural effusion was bilateral in 6 out of the 9 patients with both pericardial and pleural effusions. The association was marginally significant (P = 0.07). The volume of pleural effusion was not associated with the presence or absence of pericardial effusion; however, the volumes of pleural and pericardial effusions showed positive ordinal correlation (Spearman’s Rho = 0.62; 95% confidence interval = 0.33–0.88; P < 0.001).

Pericardial effusion was more frequently seen with a pulmonary consolidation pattern (10/25 with consolidative changes compared to 1/12 without consolidation) and with lymphadenopathy (8/20 with lymphadenopathy compared to 3/17 without lymphadenopathy); however, these associations did not reach a statistically significant value.

A follow-up CT was performed in 10 patients (8 with CT-evidence of pericardial effusion and 2 without). Pericardial effusion resolved in 3, decreased in 4, and was unchanged in 1 patient(s). Newly developed effusion was seen in 2 patients. However, the interval between the index CT and the follow-up study was highly variable with mean of 659 ± 785 days and range of 87–4054 days.

In 9 of the primary 11 patients with evidence of pericardial effusion on CT chest, an echocardiography was performed within a median of 27 days and confirmed the persistence of effusion in 5 patients. Effusion resolved in 4 patients.

## Discussion

Over the past 20 years, the incidence of coccidioidomycosis has increased [18, 19]. The infection can result in significant morbidity and requires increased awareness particularly in endemic areas where travelers may visit from non-endemic regions. Abnormalities of the lung parenchyma in the form of consolidation, nodules or cavities have been already well described [5, 20]. Other thoracic manifestations of disease such as intrathoracic lymphadenopathy and pleural effusions as well as extrapulmonary manifestations are also well known [21–24].

Pericardial disease in the setting of coccidioidomycosis has been documented only in case reports or review of such case reports in the past [15, 17] and has not been systematically evaluated. Pericardial involvement by infection may theoretically occur from contiguous spread from lung parenchyma or adjacent lymphadenopathy versus hematogenous spread. We found that pericardial imaging abnormalities were not present in most patients diagnosed with coccidioidomycosis. Most patients with pericardial effusions in our study did not have extrapulmonary dissemination. This may suggest that an adequate immune response is needed to develop a pericardial effusion.

## Conclusion

Pericardial imaging abnormalities are seen uncommonly in the context of coccidioidal infection. Pulmonary nodules or consolidation are prevalent imaging manifestations of this disease. This is the first systematic study to evaluate the pericardium in patients with coccidioidal infection with CT imaging and could be an additional tool for the diagnostic workup of patients with coccidioidomycosis or patients with suspected pericardial disease. Our study is limited since for most of our patients, the diagnosis of coccidioidal infection was made based on serology and not based on pericardial fluid sampling therefore reactive inflammatory etiology of pericardial effusion or other causes related to comorbid conditions are plausible. Additionally, about half of the CT scans in our study were noncontrast in technique which may limit our data. The study is also limited by its retrospective nature, small sample size and is based on data from a single institution. Due to its retrospective design, a cause-effect relationship cannot be determined among variables. Future studies correlating with multimodality imaging including echocardiography or magnetic resonance imaging (MRI) can be considered for evaluation of constrictive physiology associated with the pericardial effusions. MRI may even be utilized for evaluation of potential myocardial abnormalities such as edema and fibrosis with this infection. In summary, our study demonstrates that the pericardium is an infrequent site of imaging abnormality in patients with coccidioidal infection.

## Data Availability

The datasets used and/or analyzed during the current study are available from the corresponding author on reasonable request.

## Abbreviations

CT: Computed tomography
ACR: American College of Radiology
IRB: Institutional review board
PACS: Picture archiving and communication system
MRI: Magnetic resonance imaging
SOB: Shortness of breath
